# Metal Ions Sensing by Biodots Prepared from DNA, RNA, and Nucleotides

**DOI:** 10.3390/bios11090333

**Published:** 2021-09-13

**Authors:** Maofei Wang, Masaki Tsukamoto, Vladimir G. Sergeyev, Anatoly Zinchenko

**Affiliations:** 1 Graduate School of Environmental Studies, Nagoya University, Furo-cho, Chikusa-ku, Nagoya 464-8601, Japan; wang.maofei@b.mbox.nagoya-u.ac.jp; 2 Graduate School of Informatics, Nagoya University, Furo-cho, Chikusa-ku, Nagoya 464-8601, Japan; tsukamoto@i.nagoya-u.ac.jp; 3 Department of Chemistry, Lomonosov Moscow State University, 119899 Moscow, Russia; sergeyevvg@gmail.com

**Keywords:** DNA, RNA, nucleotides, hydrothermal synthesis, nanoparticles, sensing, metal ions, mercury, silver, copper

## Abstract

Nucleic acids that exhibit a high affinity toward noble and transition metal ions have attracted growing attention in the fields of metal ion sensing, toxic metal ion removal, and the construction of functional metal nanostructures. In this study, fluorescent nanoparticles (biodots) were synthesized from DNA, RNA, and RNA nucleotides (AMP, GMP, UMP, and CMP) using a hydrothermal (HT) method, in order to study their metal ion sensing characteristics. The fluorescent properties of biodots differ markedly between those prepared from purine and pyrimidine nucleobases. All biodots demonstrate a high sensitivity to the presence of mercury cations (Hg^2+^), while biodots prepared from DNA, RNA, and guanosine monophosphate (GMP) are also sensitive to Ag^+^ and Cu^2+^ ions, but to a lesser extent. The obtained results show that biodots inherit the metal ion recognition properties of nucleobases, while the nucleobase composition of biodot precursors affects metal ion sensitivity and selectivity. A linear response of biodot fluorescence to Hg^2+^ concentration in solution was observed for AMP and GMP biodots in the range 0–250 μM, which can be used for the analytic detection of mercury ion concentration. A facile paper strip test was also developed that allows visual detection of mercury ions in solutions.

## 1. Introduction

Nucleic acids form complexes with a broad variety of metal cations [1]. The fundamental properties of such complexes were intensively studied in the 1960s and 1970s [2]. Nowadays, DNA is no longer considered only as a carrier of genetic information, and the scope of DNA applications is steadily growing. At present, DNA is actively utilized for the construction of various functional systems [3,4,5] that either utilize metal ions as active components of such systems or target metal ions for detection or analysis. In particular, the strong DNA affinity for heavy metal ions [1] was used for the sensing [6,7] and removal [8,9,10] of Hg^2+^, Pb^2+^, and other toxic metal ions from water.

Nanobiosensing is a rapidly developing scientific field [11], and a vast number of materials for metal ion sensing have been prepared using the hydrothermal (HT) treatment of biomass [12], which involves the heating of aqueous solutions or dispersions of biomass, usually at 200–300 °C in an autoclave. The formation of a graphene-like type of carbon nanoparticles (carbon dots, CDs) and polymeric nanoparticles during the HT processing of biomass has been repeatedly demonstrated [12,13]. CDs produced from biomass have high fluorescence [14], low toxicity [15], and low price compared to conventional fluorescent dyes. Therefore, applications of CDs in energy [16], catalysis [17], biomedicine [18], and other fields are anticipated.

DNA is a ubiquitous macromolecule that can be extracted in large quantities from fish milt and other natural sources. The development of technologies for large-scale DNA extraction has made it possible to consider DNA as a new biomass resource [4,19,20,21], which can be used for the production of new materials, similarly to well-known biomass resources such as polysaccharides, proteins, and fats. In particular, DNA has been explored as a precursor for the preparation of fluorescent nanomaterials by HT synthesis. Several research groups have reported the conversion of DNA into fluorescent nanomaterials (biodots) [19,20,21,22]. Furthermore, the possibility of applying DNA biodots for Hg^2+^ sensing was demonstrated [20]. DNA and RNA biomacromolecules are composed of four types of monomeric nucleotides, and it is considered that the nucleotide composition should affect the fluorescent characteristics of nanoparticles prepared from nucleic acids. For instance, Guo et al. showed that the fluorescent properties of biodots prepared from individual nucleobases of DNA greatly varied [19]. Consequently, the sensing characteristics of biodots prepared from macromolecular nucleic acids (DNA and RNA) and from individual nucleotides are expected to be different; however, related studies are not found in literature, to the best of our knowledge.

Here, we systematically studied and compared the fluorescence and metal ion sensing characteristics of fluorescent nanoparticles (biodots) prepared by HT treatment of DNA, RNA, and RNA nucleotides (AMP, GMP, CMP, and UMP) at 200 °C. From a practical viewpoint, we showed that the biodots prepared in this study can be used for analytical detection of Hg^2+^ concentration in aqueous solutions, as well as for a facile visual detection of Hg^2+^, Ag^+^, Cu^2+^, and a number of other heavy metal ions, using biodot-impregnated paper strips. Water contamination by Hg^2+^ represents a serious threat to human health and causes numerous diseases such as Minamata disease [23]. Furthermore, the widespread use of silver nanoparticles as antimicrobial agents in many products is expected to cause their accumulation in the aquatic environment and the consequent release of silver cations. Silver is known to be one of the most toxic metals to organisms, showing effects at ng/L concentrations [24]. Therefore, environmental pollution with silver nanoparticles is a major issue that requires careful monitoring of Ag^+^ concentration in the environment. The simple biodot strip sensor proposed in this study would be useful for rapidly detecting contamination with such metal ions.

## 2. Materials and Methods

### 2.1. Materials

Deoxyribonucleic acid (DNA) from salmon milt (ca. 100–300 bp, purity >90%) was purchased from Fujifilm Wako (Osaka, Japan). RNA from yeast was purchased from Tokyo Chemical Industry Inc. (Tokyo, Japan). Cytidine-5′-monophosphate (purity 98%), adenosine-5′-monophasphate (purity 98%), guanosine-5′-monophasphate (purity 97%), and uridine-5′-monophasphate (purity 98%) were purchased from Combi Blocks Inc. (San Diego, CA, USA). KNO_3_, NaCl, CaCl_2_·2H_2_O, Al(NO_3_)_3_·9H_2_O, Zn(NO_3_)_2_·6H_2_O, Co(NO_3_)_2_·6H_2_O, Pb(NO_3_)_2_, NiCl_2_·6H_2_O, Cu(NO_3_)_2_·3H_2_O from Fujifilm Wako (Osaka, Japan), Hg(NO_3_)_2_·H_2_O and AgNO_3_ from Sigma-Aldrich (St. Louis, MO, USA), Cd(NO_3_)_2_·9H_2_O from Combi-Blocks (San Diego, CA, USA), and MgCl_2_·6H_2_O from Kishida Chemicals (Osaka, Japan) were used for metal ion sensing experiments, as received. Unless otherwise mentioned, deionized water of resistivity 18.2 MOhm⋅cm and purified using a Purelab Chorus 1 Life Science apparatus was used in all experiments.

### 2.2. Methods

#### 2.2.1. Fluorescence Spectroscopy (FS)

The fluorescence spectra of biodots in deionized water and in solutions containing various metal ions were recorded on an FP-6600 spectrofluorometer (Jasco, Tokyo, Japan) in 1 cm × 1 cm × 5 cm quartz cells (optical path 1 cm) at room temperature. For fluorescence measurements, a solution of biodots obtained after dialysis and diluted 100 times was used.

#### 2.2.2. Nucleic Magnetic Resonance Spectroscopy (NMR)

D_2_O solution (0.67 mL) of DNA biodots (ca. 3 mg) was transferred into a 5 mm NMR tube and ^1^H and ^31^P NMR spectra were measured on a JNM-ECA500 instrument (JEOL, Tokyo, Japan). After the measurements, 10 μL of CH_3_CN–D_2_O (10% *v*/*v*) was added to the above NMR tube and ^13^C NMR was measured. Chemical shifts of ^1^H, ^31^P, and ^13^C NMR in D_2_O are expressed in parts per million (ppm) relative to HDO at δ 4.79 (at 23.9 °C), [25] external 85% H_3_PO_4_ at δ 0.00, and a trace amount of CH_3_CN at δ 1.47 [25], respectively.

#### 2.2.3. Transmission Electron Microscopy (TEM)

TEM observations were performed using a JEM-2100 Plus microscope (JEOL) at 200 kV acceleration voltage. A drop of a solution containing biodots of ca. 5% (*w*/*v*) was placed onto a carbon film (10 nm) coated TEM grid (Alliance Biosystems, Osaka, Japan). The solution was removed after 5 min of deposition with a filter paper, and the grid was dried in a dry box at relative humidity <10% overnight before TEM observation.

### 2.3. Hydrothermal Synthesis of Biodots

Sodium salts of nucleic acids and nucleotides were directly dissolved in Millli-Q water at 1% (*w*/*v*) concentration. Nucleotides received as acids were dissolved in water containing equimolar concentrations of NaOH. Then, 10 mL of 1% (*w*/*v*) solution of a nucleic acid was transferred to 25 mL polytetrafluoroethylene cup that was placed into a stainless autoclave reactor vessel HU-25 (SAN-AI Kagaku, Nagoya, Japan) and tightly closed. The autoclaved reactor was then heated in a convection oven at 200 °C for 10 h under autogenous pressure. After HT treatment, the reaction mixture was chilled to ambient temperature and a precipitate (if present) was separated by centrifugation at 4000 rpm for 10 min. To remove low-molecular-weight products, the resulting solution was dialyzed against 500 mL deionized water using 3 mL Slide-A-Lyzer dialysis cassettes (Thermo Fisher Scientific, Waltham, MA, USA) with molecular weight cut-off (MWCO) 2000 Da twice for 2 h and once for 6 h. The solutions of biodots were stored in a refrigerator at 4 °C. Yields of biodots synthesized from RNA and nucleotides were ca. 20% and that of biodots synthesized from DNA was ca. 30%, as reported in detail elsewhere [26].

### 2.4. Preparation of Paper-Based Strips with Biodots for Metal Ions Detection Test

Paper strips of ca. 1 cm × 5 cm size were immersed in water solutions of biodots of ca. 1% (*w*/*v*) concentration three times each for 2–3 s, then removed and air-dried in a convection oven at 40 °C for 30 min. The paper strips containing biodots were dipped into solutions of various metal ions for 2–3 s, air-dried, and their photographic images were taken under 365 nm UV irradiation. No detectable release (<0.01%) of biodots was measured by fluorescence spectroscopy after the dipping of strips into solutions of metal ions.

## 3. Results

### 3.1. Synthesis of Biodots from Nucleic Acids

**Figure 1:**
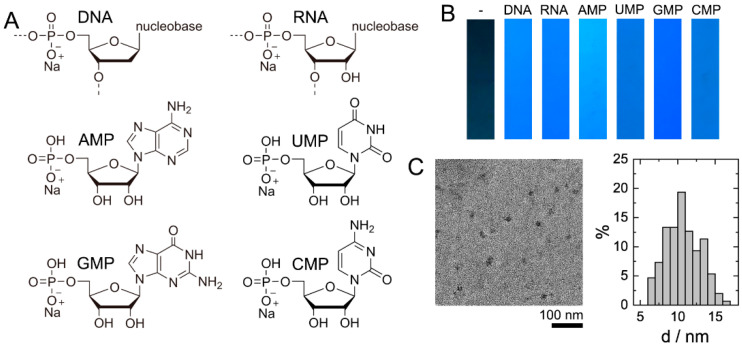
Fluorescent biodots prepared from nucleic acid. (**A**) Chemical structures of DNA, RNA, sodium adenosine monophosphate (AMP), sodium uridine monophosphate (UMP), sodium guanidine monophosphate (GMP), and sodium cytosine monophosphate (CMP) used for biodot synthesis. (**B**) Photographic images of paper strips impregnated with biodots prepared from DNA, RNA, AMP, UMP, GMP, and CMP using a soaking-drying method under irradiation with 365 nm UV light. The control paper strip is marked with (-). (**C**) Typical transmission electron microscopy images of biodot nanoparticles prepared by HT treatment of DNA and their size distribution. A total of 150 particles were measured to build the distribution.

DNA from salmon sperm (ca. 100–300 bp), RNA from yeast, and four nucleotides of RNA: sodium adenosine monophosphate (AMP), sodium cytosine monophosphate (CMP), sodium guanidine monophosphate (GMP), and sodium uridine monophosphate (UMP) (Figure 1A) were converted into a fluorescent material by HT treatment of their 1.0% solutions at 200 °C for 10 h. Optimization of temperature and HT treatment time parameters was performed and discussed in the preceding study [26].

HT treatment of each type of nucleic acid yielded a material (biodots) showing strong fluorescence under UV irradiation (Figure 1B). The fluorescent intensity, as well as color of the light emitted by the biodots, were markedly dependent on the chemical structure of their precursors (Figure 1B). In particular, there was a notable difference in brightness of fluorescent materials prepared from nucleotides containing purine (AMP and GMP) and pyrimidine (UMP and CMP) nucleobases, where the former emitted a stronger fluorescence. The product of DNA HT treatment contained nanoparticles of size 10.7 ± 2.4 nm, (Figure 1C) which was of the same order as the DNA-derived nanoparticles reported by Song et al. [20] and Ding et al. [22]. Fluorescent nanoparticles were purified from molecular impurities by dialysis through a separation membrane having a molecular weight cut-off of 2000 Da.

**Figure 2:**
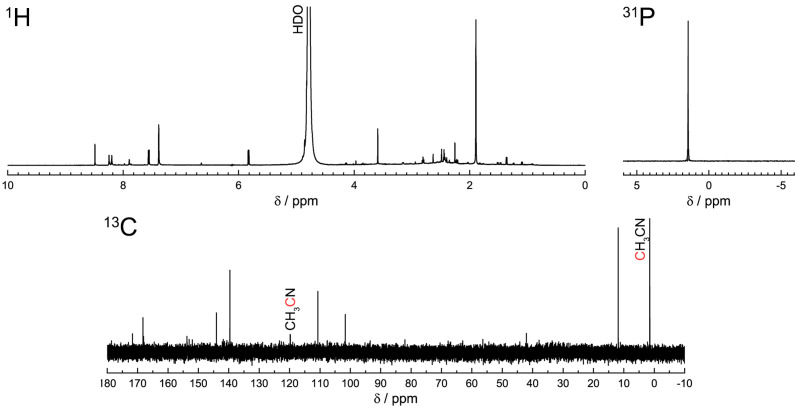
Biodots characterization by NMR spectroscopy. ^1^H, ^13^C, and ^31^P NMR spectra of DNA biodots in D_2_O after dialysis.

NMR spectroscopy analysis of biodots after dialytic purification indicated the decomposition of the ribose part, evident from the disappearance of ribose signals at 4–5 ppm in the ^1^H NMR spectrum and relevant signals at 50–60 ppm in the ^13^C NMR spectrum (Figure 2). The aromatic part remained and its signals appeared at 7–9 ppm in the ^1^H NMR spectrum and at 100–150 ppm in the ^13^C NMR spectrum. The ^31^P NMR spectrum contained a single signal at 1.7 ppm, indicating the presence of the phosphate in the biodots. Based on NMR data and the analysis of DNA structural changes under high temperatures reported earlier [27,28,29], it was concluded that nucleobases play a primary role in the process of biodot formation. Key reactions in this process are the release of nucleobases from nucleic acids by depurination and depyrimidation mechanisms [30,31] and their consequent condensation by the reaction of alkyliminodeoxy substitution [32]. The condensation of carbonyl and amino groups of nucleobases can lead to the polymerization and crosslinking of nucleobases [32]. The formation of conjugated aromatic systems in this process determines the strong fluorescence of the product, whereas the hydrophobicity of such macromolecules causes their assembly into nanoparticles (biodots).

**Figure 3:**
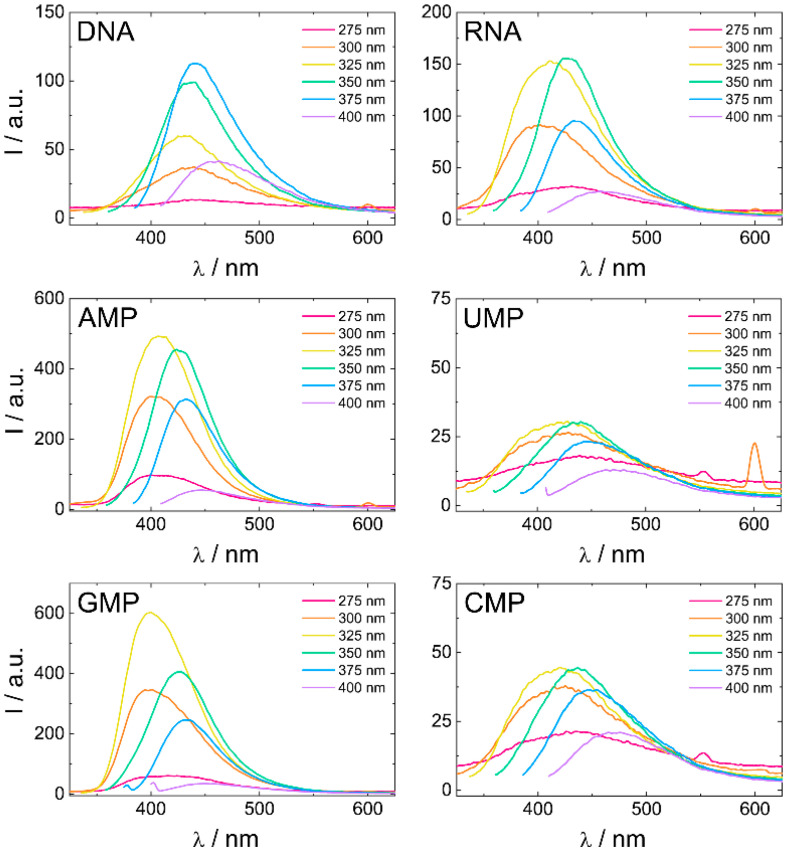
Comparison of biodot fluorescent properties. Fluorescence spectra of biodot solutions of ca. 70 mg/L concentration and at pH = 7.5 prepared from DNA, RNA, and nucleotides after dialysis at excitation wavelengths λ_ex_ = 275–400 nm.

Fluorescence spectra of biodots at excitation wavelengths λ_ex_ = 275–400 nm are compared in Figure 3. As was expected based on the visual observation of biodot-impregnated paper strips (Figure 1B), the fluorescence of the biodots varied markedly. The fluorescence intensities of individual biodots at different excitation wavelengths were reproducible within a ±10–15% deviation range (Supporting Information, Figure S1). Most importantly, there was a notable difference (ca. 15–20 times) between the fluorescence of the brighter biodots prepared from purine nucleotides (AMP and GMP) and those prepared from pyrimidine nucleotides (UMP and CMP). The fluorescence of biodots prepared from polymeric RNA containing AMP, GMP, UMP, and CMP in its structure was somewhat lower than the average fluorescence of biodots prepared from individual nucleotides. The Fluorescence intensity of biodots prepared from DNA and RNA, which are distinct by a minor difference in the structure of their sugar parts and the chemical structure of one nucleobase (RNA’s uridine (U) vs. DNA’s thymine (T)), differed about two-fold, and the RNA biodots had a brighter fluorescence. One can also note that the strongest fluorescence of RNA dots was emitted under 325–350 nm excitation, which is similar to the biodots of all RNA nucleotides, but different from DNA biodots, having the strongest fluorescence at longer excitation wavelengths (350–370 nm). The difference in biodot fluorescence is due to different quantum yields, which greatly vary, from several percent for weakly fluorescent CMP and UMP biodots, to up to 30% for strongly fluorescent AMP biodots [26].

### 3.2. Chemical Sensing of Metal Ions by Biodots

**Figure 4:**
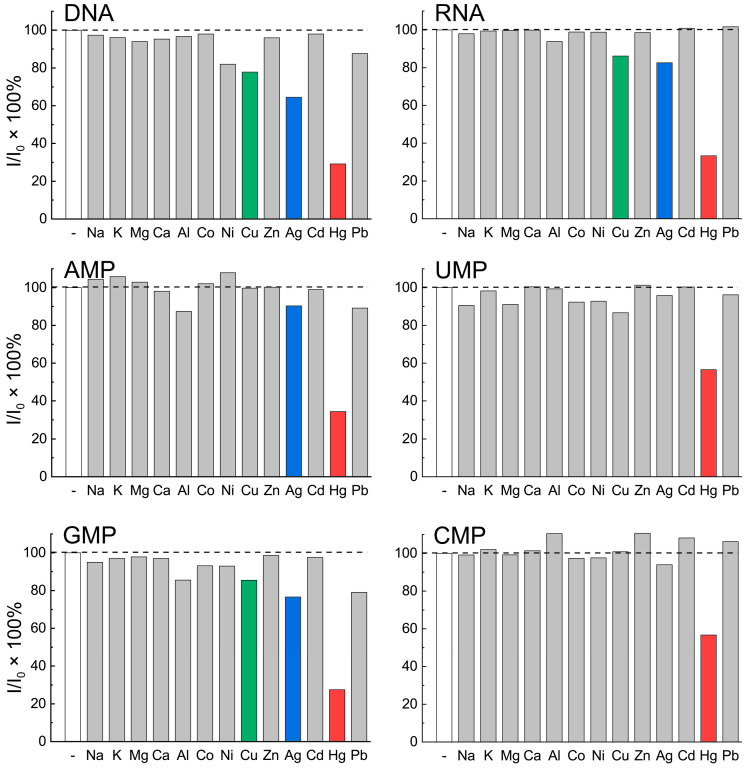
Sensing of metal ions by biodots. Comparison of normalized fluorescence intensities (%) of biodots of ca. 20 mg/L concentration prepared from DNA, RNA, AMP, GMP, UMP, and CMP at pH = 7.5 in the presence of various metal cations at 330 μM concentration. Excitation and emission wavelengths were chosen as follows: DNA (λ_ex_ = 350 nm, λ_em_ = 435 nm), RNA (λ_ex_ = 325 nm, λ_em_ = 402 nm), AMP (λ_ex_ = 325 nm, λ_em_ = 405 nm), UMP (λ_ex_ = 325 nm, λ_em_ = 422 nm), GMP (λ_ex_ = 325 nm, λ_em_ = 397 nm), and CMP (λ_ex_ = 325 nm, λ_em_ = 415 nm).

The strong affinity for nucleic acids for a broad variety of metal ions suggests the possibility of using biodots for metal ion detection [7]. Furthermore, considering the dramatic dependence of biodot fluorescent characteristics on the structure of the starting material used for HT synthesis (Figure 3), the sensitivity of different biodots toward metal ions was also expected to alter. For a quantitative comparison of the fluorescence properties of DNA biodots, the fluorescent intensities of biodots were measured in solutions of 13 types of metal cations, at concentrations of 330 μM (Figure 4). The biodots used for these measurements were purified by dialysis against a 2000 Da membrane to remove low-molecular weight impurities; however, the dialytic purification of biodots was found to be non-critical, as the sensing characteristics of the prepared biodots were similar to those of the dialyzed ones.

The biodots exhibited a distinct response to a number of specific metal ions (Figure 4). The fluorescence intensity of purified biodots obtained from DNA and other nucleic acid precursors was practically independent of the presence of alkali or alkaline earth metal ions (I/I_0_ > 95%). Even at 0.1 M NaCl concentration, the fluorescence intensity of biodots changed by less than 1% (data not shown). The presence of transition metal ions in solutions caused quenching of the biodots’ fluorescence, and the degree of quenching varied greatly among the studied metal ions. The most efficient quenching of DNA and RNA biodot fluorescence, by ca. 70%, under the studied conditions was measured for Hg^2+^ ion. DNA biodots also showed a notable sensitivity to Ag^+^ ions and a somewhat weaker sensitivity to Ni^2+^, Cd^2+^, and Pb^2+^ ions. RNA biodots were only sensitive to Ag^+^ and Cu^2+^ ions, but the sensitivity was much weaker in comparison to Hg^2+^. It can be noted that, whereas a high sensitivity of DNA biodots to Hg^2+^ ion was observed, their response to other toxic metal ions (Ag^+^, Cu^2+^) could be used to create multi-target sensing platforms.

In contrast to biodots of DNA and RNA, biodots derived from nucleotides showed a higher sensitivity to Hg^2+^ ions. (Figure 4). Biodots prepared from nucleotides with purine nucleobases (AMP and GMP) exhibited stronger fluorescence quenching in the presence of mercury ions compared to biodots from pyrimidine ones (CMP and UMP). AMP and GMP biodots differed in the manner that GMP biodots were sensitive to Hg^2+^, Ag^+^, and Cu^2+^, similarly to DNA and RNA, whereas the AMP biodots showed a response only to Hg^2+^ ions. The sensitivity of DNA and RNA biodots to Ag^+^ and Cu^2+^ is thus ascribed to the presence of guanine nucleobases in the corresponding macromolecules. UMP and CMP biodots exhibited almost identical sensing behaviors, with a moderate, but very selective, sensitivity to Hg^2+^ ions. Note that, due to the high sensitivity of biodots to Hg^2+^, the degree of their fluorescence quenching by Hg^2+^ is not affected by the coexistence of other ions, even at 30 times higher concentrations (Supporting Information, Figure S2).

**Figure 5:**
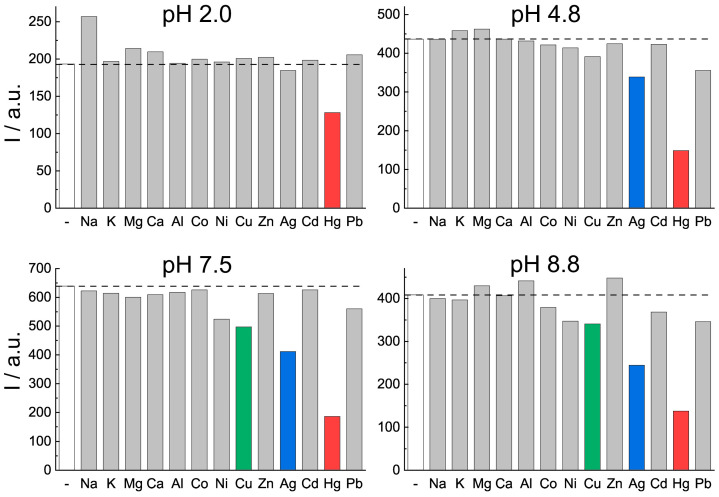
Metal ion sensing by DNA biodots at different pHs. Fluorescence intensities of DNA biodots of ca. 20 mg/L concentration in solutions of various metal ions at 330 μM concentration and at different solution pHs. Fluorescence intensity was measured at λ_em_ = 435 nm upon excitation at λ_ex_ = 350 nm.

To address the application of biodots for metal sensing in biological and environmental samples, the sensing characteristics of DNA biodots were studied in solutions of different pHs, from 2 to 12 (Figure 5). The addition of alkali and alkali-earth metal ions and Al^3+^ did not affect the biodot’s fluorescence in the whole range of studied pHs, from 2 to 12. In contrast, the fluorescence of biodots in the presence of transition metal ions, including Hg^2+^, Ag^+^, and Cu^2+^, was pH dependent. Due to the limited solubility in alkaline solutions, all transition metal ion precipitation and spectroscopic data for pH 12 are not shown. The sensitivity of biodots for transition metal ions was very similar at pH 7 and 9, except for Ag^+^, where the quenching degree slightly decreased at pH 7. At pH 5, DNA biodots were only sensitive to Hg^2+^, Ag^+^, and Pb^2+^ ions. Finally, the degree of fluorescence quenching decreased dramatically at pH 2 for all metal ions, and Hg^2+^ was the only ion that caused a decrease of DNA biodot fluorescence. The observed changes indicate the competition of H^+^ with metal ions for binding with biodots. This competition is usually expected, given that amines, N atoms of nucleotides, which are the binding sites of the metal ions under study, are protonated in acidic media. From the applied point of view, the observed behavior of the biodots suggests the possibility of adjusting the selectivity of biodots by adjusting the pH of the analyte solution and, in turn, designing multitarget sensing systems.

**Figure 6:**
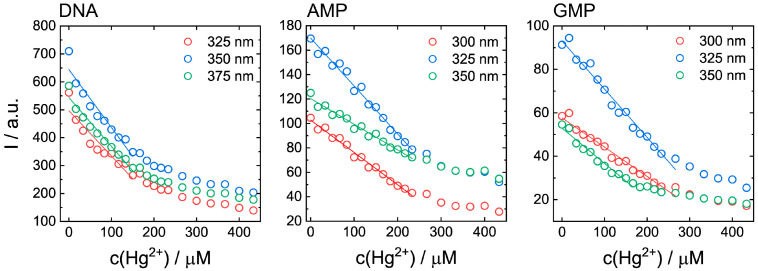
Fluorescence intensities of DNA, AMP, and GMP biodots of ca. 20 mg/L concentrations on Hg^2+^ concentration measured at the wavelengths of maximum fluorescence under different excitation wavelengths.

Considering an application of biodots for the analytical determination of Hg^2+^ concentration, we examined the correlation between Hg^2+^ concentration and fluorescence intensity of DNA biodots (Figure 6). With the addition of Hg^2+^ into the DNA biodot solution, the intensity of biodots decreased gradually until ca. 30% residual fluorescence, reaching a plateau and indicating that a part of the fluorophores on the biodots were not quenched by Hg^2+^. Regardless of the excitation wavelength, no linear correlation between Hg^2+^ concentration and DNA biodot fluorescence intensity was observed. The analogous data for AMP and GMP biodots showed that linear correlations between Hg^2+^ concentration and biodot fluorescence intensities were found in the range of Hg^2+^ concentrations between 0–220 μM for AMP biodots and between 0–250 μM for GMP biodots. Changes in excitation wavelength did not significantly affect the linearity of the dependences in the studied ranges of concentrations.

**Figure 7:**
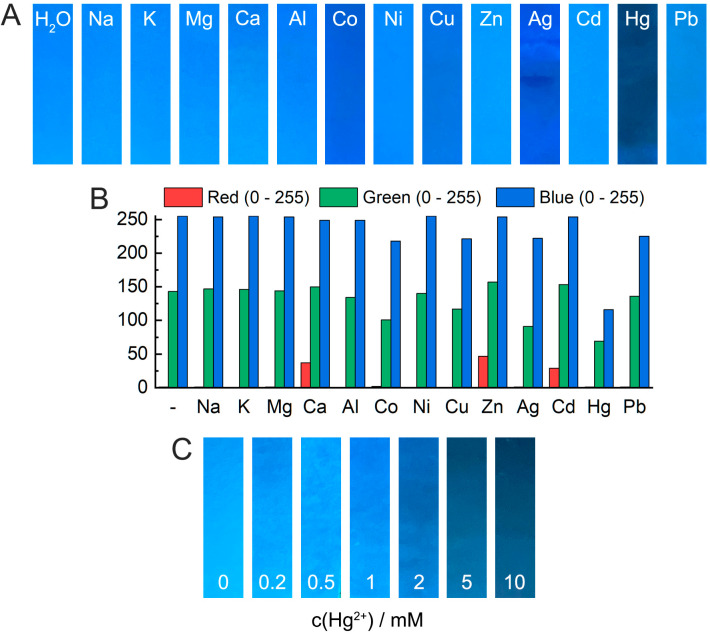
Strip test based on biodots. (**A**) Photographic images of paper strips impregnated with DNA biodots after soaking in solutions of different metal cations with concentrations of 10 mM under 365 nm UV irradiation. The master photograph is given as Supporting Information, Figure S3. (**B**) Distributions of RGB component values of the strips are shown in (**A**). (**C**) Photographic images of paper strips impregnated with DNA biodots after soaking in solutions of Hg^2+^ of different concentrations under 365 nm UV irradiation. The master photograph is given as Supporting Information, Figure S4.

The quenching of biodot fluorescence by transition metal cations shown above can be applied for the visual detection of Hg^2+^ and several other metal ions, using biodots as an indicator. To test this idea, we developed a simple paper strip test for metal ions. Paper strips were impregnated with biodots by a facile soaking–evaporation treatment. Photographic images of paper-strips with DNA biodots after soaking with solutions of different metal ions are shown in Figure 7. Essentially, no difference from the control (H_2_O) sample was observed after testing alkali and alkali-earth cations. The fluorescence of the paper strips with biodots was quenched most efficiently by Hg^2+^ and to a significant extent by Co^2+^, Cu^2+^, and Ag^+^, in reasonable agreement with the data in Figure 4. The shade of strips with quenched fluorescence also varied, and appeared as navy for Hg^2+^, sapphire blue for Ag^+^ and Co^2+^, azure for Cu^2+^, etc. Color analysis using the RGB color model (Figure 7B) indicated that the quenching of biodot fluorescence by metal ions results in a decrease of blue and green components of the original fluorescence color of strips, and the degree of each change affects the resulting shade. It should be mentioned that the order of biodot fluorescence quenching efficiency by metal ions in the paper strip test (Figure 7A) does not exactly correspond to the spectroscopic data (Figure 4); for instance, for Co^2+^ and Ni^2+^ cations. This could be caused by factors related to the state of the biodots in solution and in the cellulosic matrix of the paper strips, different water and ion activities, differences in the excitation wavelengths, etc. The proposed strip test has the highest sensitivity to Hg^2+^ cations; therefore, even lower concentrations of Hg^2+^ can be detected (Figure 7C). A strip test could be potentially used for the quick detection of heavy metal ion pollution in environmental samples, but the sensitivity of the strips toward metal ions should be further improved, in order to be able to detect mercury pollution at ppb level.

## 4. Discussion

The high sensitivity of biodots to Hg^2+^ (Figure 4, Figure 5 and Figure 7) apparently originates from the intrinsic strong affinity of nucleobases to mercury ions [33,34,35]. The strong coordination of Hg^2+^ with thymine [36] and uracil [37] nucleobases was reported to be the predominant binding mechanism, yet complexes of adenine and cytosine with Hg^2+^ were also reported [38,39]. The strong biding of nucleic acids with Ag^+^ and Cu^2+^, which involves binding with nucleobases, is also known [1], and this affinity was utilized for the metallization of DNA macromolecules to construct Ag [40] and Cu [41] nanowires and Ag and Cu nanoparticle arrays [42,43]. The major role of nucleobases in biodot formation during HT treatment, which was revealed by NMR data (Figure 2), implies the inheritance of DNA recognition properties toward metal ions by biodots, and this was in accordance with the experimentally observed sensing preferences of biodots to Hg^2+^, Ag^+^, and Cu^2+^.

The higher sensitivity of AMP and GMP biodots to these metal ions, compared to UMP and CMP (Figure 4), can be explained by taking into account the possible binding mechanisms. For the formation of stable complexes such as T-Hg-T and U-Hg-U, the coordination of Hg^2+^ with two imines of nucleobases is required [44]. Based on the proposed mechanism of biodot formation [26,32], the probability of such a coordination between Hg^2+^ and two imines of biodots is low, and it is expected that Hg^2+^ mainly interacts with either a single imine or single amino group. Since AMP and GMP biodots have more nitrogen-containing groups than UMP and CMP biodots, this, apparently, determines the stronger binding of AMP and GMP biodots to Hg^2+^ and, as a consequence, the stronger quenching of biodot fluorescence. The same explanation is valid for the higher sensitivity of AMP and GMP biodots to Ag^+^ and Cu^2+^, which have coordination sites similar to Hg^2+^.

The higher selectivity of AMP, CMP, and UMP biodots to Hg^2+^ ion compared to RNA biodots is ascribed to the effect of the chemical homogeneity of nucleic acids used for HT synthesis. In other words, HT treatment of macromolecular DNA or RNA results in a broad variety of chemical transformations, caused by the presence of four monomeric building blocks (nucleotides) in the DNA and RNA structure. Consequently, this originates a broad spectrum of metal ion binding sites, with different binding characteristics. As a result, the fluorescence of DNA and RNA biodots is affected not only by Hg^2+^ ions but also by several other ions: Ag^+^, Cu^2+^, Ni^2+^, and Pb^2+^ (Figure 4). In contrast, HT treatment of individual nucleotides produces more chemically homogenous biodots with a limited variation of binding centers, which results in their higher selectivity to only a few metal ions; i.e., exclusively to Hg^2+^ ion by AMP, CMP, and UMP biodots, and to Hg^2+^, Ag^+^, and Cu^2+^ by GMP biodots. The aforementioned chemical homogeneity can also be the reason for the better linear correlation between AMP and GMP biodot fluorescence and Hg^2+^ concentration compared to biodots prepared from DNA.

## 5. Conclusions

Fluorescent nanoparticles (biodots) synthesized from nucleic acids inherit the high affinity of nucleic acids to Hg^2+^ and, to a lesser extent, to Ag^+^ and Cu^2+^, which was utilized for the sensing of these heavy metal ions in aqueous solutions, as well as for naked eye detection of these ions. The fluorescent properties and metal ion sensing characteristics of biodots are notably affected by the chemical structure of the nucleic acid used as a starting material for HT treatment. Fluorescent nanoparticles prepared from individual AMP and GMP nucleotides exhibit a significantly higher fluorescence compared to biodots prepared from UMP, CMP, and DNA. Furthermore, biodots prepared from individual nucleotides show better selectivity to Hg^2+^ ions and a linear dependence of fluorescence on Hg^2+^ concentration, in a broad concentration range. In addition, biodots can be used to make paper-based sensor strips for detecting metal ions in aqueous solutions. Paper strip-based sensors represent a new strategy for developing simple metal ion indicators for a variety of analytical and environmental applications.

## Data Availability

Not applicable.

## Supplementary Materials

The following are available online at https://www.mdpi.com/article/10.3390/bios11090333/s1. Figure S1: Reproducibility of fluorescent characteristics of DNA biodots; Figure S2: Sensitivity of DNA biodots to Hg^2+^ in the presence of background cations; Figures S3 and S4: Original photographs of biodots-impregnated strips shown in Figure 7A, C.
