# Supplementary material for: Metal Ions Sensing by Biodots Prepared from DNA, RNA, and Nucleotides
Source: Biosensors (Basel). 2021 Sep 13;11(9):333. doi: 10.3390/bios11090333 (PMC8466223; doi:10.3390/bios11090333)
Supplement: Supplementary file 1 [file biosensors-11-00333-s001.zip › biosensors-1326929-supplementary.pdf]

Supplementary Material

Metal Ions Sensing by Biodots Prepared from DNA, RNA, and Nucleotides

Maofei Wang,[1] Masaki Tsukamoto,[2] Vladimir G. Sergeyev,[3] Anatoly Zinchenko[1,*]

1 Graduate School of Environmental Studies, Nagoya University, Furo-cho, Chikusa-ku, Nagoya 464-8601, Japan; wang.maofei@b.mbox.nagoya-u.ac.jp

2 Graduate School of Informatics, Nagoya University, Furo-cho, Chikusa-ku, Nagoya 464-8601, Japan; tsukamoto@i.nagoya-u.ac.jp

3 Department of Chemistry, Lomonosov Moscow State University, Moscow 119899, Russia; sergeyevvg@gmail.com

* Correspondence: zinchenko@urban.env.nagoya-u.ac.jp; Tel.: +81-52-789-4771

1. Reproducibility of fluorescent characteristics of DNA biodots.

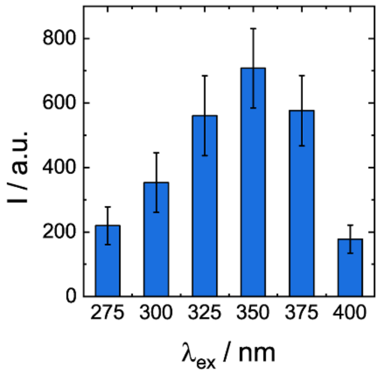

Figure S1. Average values and standard deviations of fluorescence intensities of DNA biodots that were prepared during three independent runs of HT treatment and biodots purification by dialysis under the same conditions (HT treatment at 200°C for 10 hours and dialysis against membrane with molecular weight cut-off (MWCO) 2000 Da twice for 2 h and once for 6 h.

2. Sensitivity of DNA biodots to Hg^{2+} in the presence of background cations.

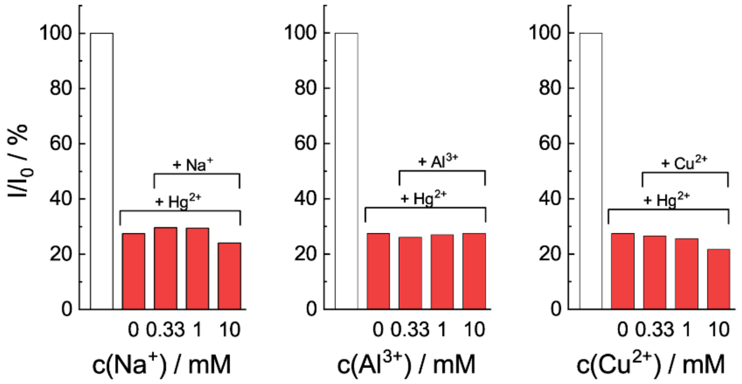

Figure S2. Comparison of normalized fluorescence intensities of DNA biodots in the presence of Hg^{2+} ions at 330 μM concentration and background ions (Na^{+}, Al^{3+}, and Cu^{2+}) at various concentrations. Excitation and emission wavelengths were λ_{ex} = 350 nm, λ_{em} = 435 nm, respectively. White bars correspond to the original fluorescence intensities of DNA biodots solutions (pH = 7.5) without cations.

3. Original photographs of biodots-impregnated paper strips.

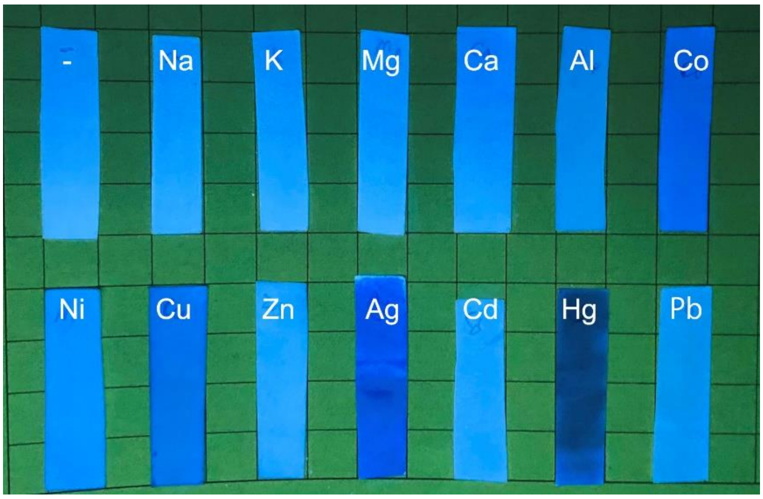

Figure S3. The original image of paper strips impregnated with DNA biodots after soaking in solutions of different metal cations of 10 mM concentration and drying under 365 nm UV irradiation.

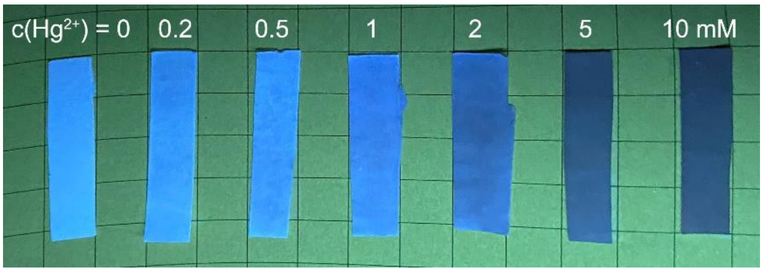

Figure S4. The original image of paper strips impregnated with DNA biodots after soaking in solutions of Hg^{2+} of different concentrations and drying under 365 nm UV irradiation.
